# Crystal structure and photoluminescent properties of a new Eu^III^–phthalate–acetate coordination polymer

**DOI:** 10.1107/S2056989022004339

**Published:** 2022-04-28

**Authors:** Prakottakarn Jittipiboonwat, Thammanoon Chuasaard, Apinpus Rujiwatra

**Affiliations:** a Department of Chemistry, Faculty of Science, Chiang Mai University, Chiang Mai, 50200, Thailand; b Materials Science Research Center, Faculty of Science, Chiang Mai University, Chiang Mai 50200, Thailand

**Keywords:** coordination polymer, lanthanide, phthalate, acetate, crystal structure, photoluminescence

## Abstract

A new coordination polymer, poly[(acetato)aqua(μ_3_-phthalato)europium(III)], [Eu(C_8_H_4_O_4_)(CH_3_O_2_)(H_2_O)]_*n*_ or [Eu^III^(phth)(OAc)(H_2_O)] (phth^2−^ = phthalate and OAc^−^ = acetate) was synthesized and characterized, revealing it to be a supra­molecular assembly of one-dimensional [Eu^III^(phth)(OAc)(H_2_O)] chains. Each chain is built up of edge-sharing distorted tricapped trigonal–prismatic *TPRS*-{Eu^III^O_9_} building motifs and assembled in a regular fashion through hydrogen-bonding and aromatic π–π inter­actions. The fully deprotonated phth^2−^ ligand was shown to be an effective sensitizer, promoting the characteristic ^5^ *D*_0_→^7^ *F_J_* (*J* = 1–4) emissions of Eu^III^ even in the presence of the non-sensitizing OAc^−^ group.

## Abstract 2

The crystal structure of a new one-dimensional [Eu^III^(phth)(OAc)(H_2_O)] coordination polymer and its room-temperature photoluminescent properties are reported.

## 1. Chemical context

Inter­est in crystal engineering of lanthanide coordination polymers has been driven by the unique coordination chemistry and electronic properties of trivalent lanthanides (*Ln* ^III^), which bring about a wide variety of potential applications ranging from, for instance, luminescence sensing (Hasegawa & Kitagawa, 2022 ▸), magnetism (Hu *et al.*, 2021 ▸), catalysis (Sinchow *et al.*, 2021 ▸), gas storage and separation (Li & Chen, 2014 ▸), to drug delivery (Wei *et al.*, 2020 ▸) and biomolecular imaging (Miller *et al.*, 2016 ▸). However, the high coordination numbers, flexible coordination geometries and lack of directionality of *Ln*—O bonds complicate prediction of the designed polymeric frameworks, which are also greatly influenced by differences in synthetic parameters, *i.e.* reaction temperature and time, solvent, pH of reaction, *etc* (Bünzli, 2014 ▸; Qiu & Zhu, 2009 ▸). The study of structure–property relationships, which is an essence of property design, is consequently limited. 
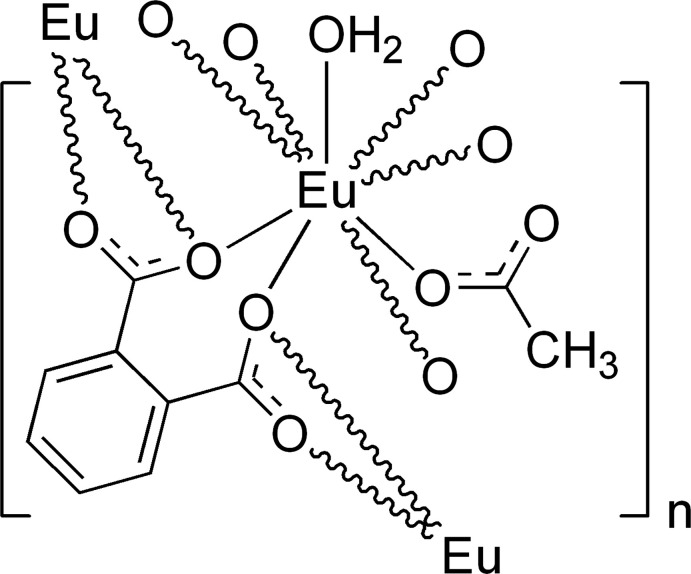


**Figure 1:**
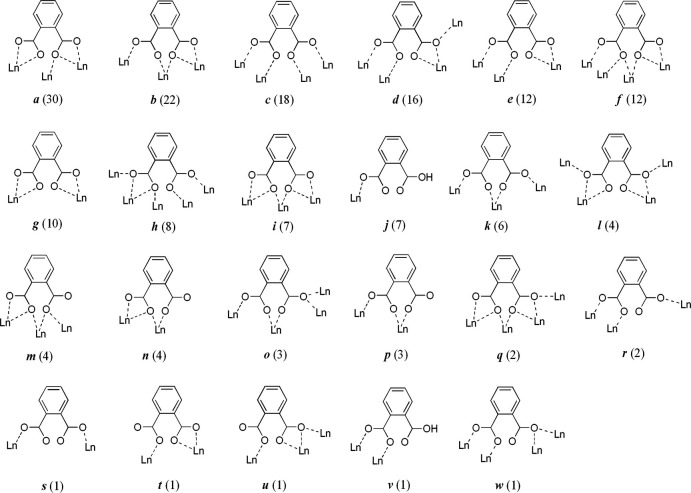
Coordination modes of phth^2−^ and Hphth^−^ found in lanthanide coordination compounds deposited to the CSD (Groom *et al.*, 2016 ▸) with frequency of appearance in parentheses.

Unlike transition-metal-based coordination polymers in which the preferred coordination geometries of the transition-metal ions play an important role in directing the framework architecture (Kitagawa *et al.*, 2004 ▸), those based on *Ln* ^III^ are principally governed by the organic ligands. Polycarb­oxy­lic acids are notably the most commonly utilized, facilitating diversity through their modes of coordination such as those found for phthalic acid (H_2_phth) (Fig. 1 ▸). These coordination modes can also be diversified through the presence of the other ligands such as those found in, for instance, [*Ln^I^* ^II^(bdc)_0.5_(phth)(H_2_O)_2_] (*Ln* ^III^ = Eu^III^, Tb^III^, Ho^III^, Er^III^ and Tm^III^, H_2_bdc = terephthalic acid; Chuasaard *et al.*, 2020 ▸), [*Ln* ^III^(abdc)_0.5_(phth)(H_2_O)_2_]·2H_2_O (*Ln* ^III^ = Eu^III^, Gd^III^ and Tb^III^, H_2_abdc = azo­benzene-4,4′-di­carb­oxy­lic acid; Chuasaard *et al.*, 2022 ▸) and [*Ln* ^III^(ox)(phth)(H_2_O)_2_]·0.5H_2_O (*Ln* ^III^ = Sm^III^ and Tb^III^, H_2_ox = oxalic acid; Wang *et al.*, 2010 ▸).

With respect to photoluminescence, phth^2−^ is acknowledged as a good sensitizer and can effectively promote *f*–*f* emissions in, for example, [Eu^III^_2_(phth)_3_(H_2_O)] (Wan *et al.*, 2002 ▸). The apparent photoluminescence can, nonetheless, be modulated by the other ligands such as ad^2−^ in [*Ln* ^III^(ad)_0.5_(phth)(H_2_O)_2_] (Chuasaard *et al.*, 2018 ▸) and bdc^2−^ in [*Ln* ^III^(bdc)_0.5_(phth)(H_2_O)_2_] (Chuasaard *et al.*, 2020 ▸).

## 2. Structural commentary

**Figure 2:**
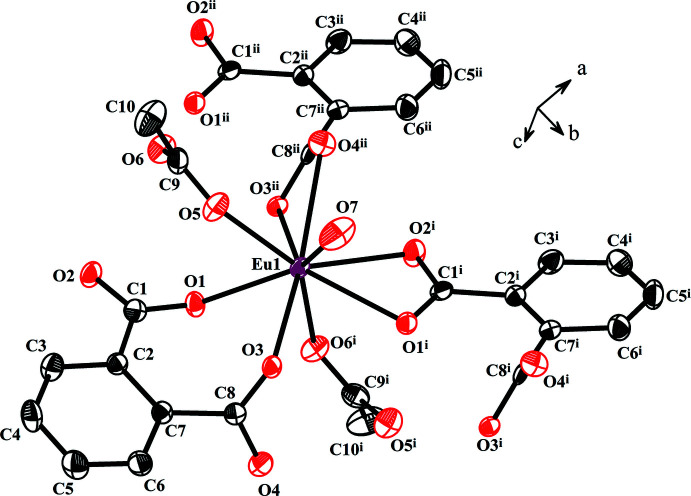
Extended asymmetric unit of the title compound drawn using 50% probability for ellipsoids (hydrogen atoms are omitted for clarity). Symmetry codes: (i) 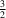 − *x*, 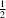 + *y*, 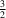 − *z;* (ii) 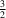 − *x*, − 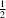 + *y*, 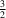 − *z*.

The asymmetric unit of the title compound, [Eu^III^(phth)(OAc)(H_2_O)], is composed of one crystallographically unique Eu^III^ ion, a whole mol­ecule of phth^2−^, and the coordinating OAc^−^ and water mol­ecules (Fig. 2 ▸). The Eu^III^ ion is ninefold coordinated to O atoms from three phth^2−^, two OAc^−^ and one water mol­ecule, which define a distorted tricapped trigonal–prismatic *TPRS*-{Eu^III^O_9_} building motif. The Eu—O bond distances are in the range 2.352 (2)-2.605 (2) Å (Table 1 ▸), which are consistent with those reported for other Eu^III^ frameworks of phth^2−^ and OAc^−^, *viz.* [Eu^III^(abdc)_0.5_(phth)(H_2_O)_2_]·2H_2_O (Chuasaard *et al.*, 2022 ▸), [Eu^III^(phth)(STP)] (NaSTP = sodium 2-(2,2′:6′,2′′-terpyridin-4′-yl)benzene­sulfonate; Hu *et al.*, 2019 ▸) and [C_2_mim]_2_[Eu_2_(OAc)_8_] (C_2_mim = 1-ethyl-3-methyl­imidazolium; Bousrez *et al.*, 2021 ▸). The *TPRS*-{Eu^III^O_9_} motifs are fused through the *μ*_2_-O atoms of phth^2−^, forming an infinite one-dimensional zigzag chain of edge-sharing *TPRS*-{Eu^III^O_9_} motifs extending along the *b*-axis direction. Not only phth^2−^, which helps facilitating the formation of the one-dimensional chain through the overall μ_3_-η^1^:η^2^:η^2^:η^1^ mode of coordination (mode *i* in Fig. 1 ▸), but also the smaller OAc^−^ link adjacent Eu^III^ centers in a bridging μ_2_-η^1^:η^1^ coord­ination mode.

## 3. Supra­molecular features

**Figure 3:**
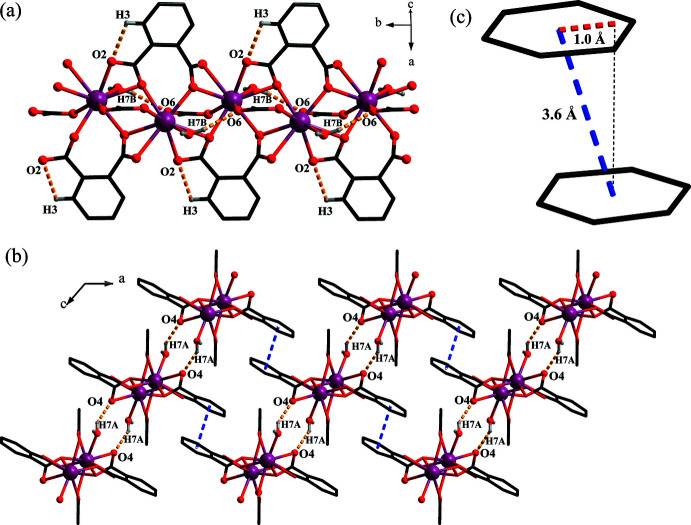
Depiction of (*a*) intra­chain and (*b*) inter­chain hydrogen-bonding inter­actions, and (*c*) π–π inter­actions.

The three-dimensional supra­molecular assembly of [Eu^III^(phth)(OAc)(H_2_O)] chains are facilitated by hydrogen bonding and aromatic π–π inter­actions (Fig. 3 ▸). The hydrogen-bonding inter­actions can be divided into the inter­chain O7—H7*A*⋯O4 and the intra­chain O7—H7*B*⋯O6 and C3—H3⋯O2 inter­actions (Table 2 ▸). The π–π inter­action between neighboring chains is considered to be of the displaced-stacking type (Banerjee *et al.*, 2019 ▸; Yao *et al.*, 2018 ▸), with an inter­planar angle of 0°, an offset distance of *ca* 1.0 Å and a centroid-to-centroid distance of *ca* 3.6 Å.

## 4. Photoluminescent properties

**Figure 4:**
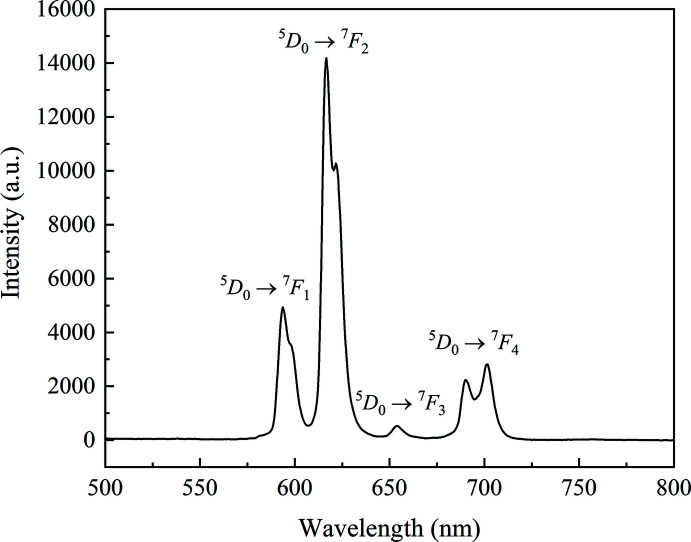
Room-temperature photoluminescent emission spectrum of the title compound.

The emission spectrum of ground crystals of the title compound was recorded at room temperature. Upon the excitation at 370 nm, the characteristic red emission originating from the ^5^ *D*_0_→^7^ *F_J_* (*J* = 1–4) transitions of Eu^III^ were displayed (Fig. 4 ▸). This indicates the efficiency of phth^2−^ as a good sensitizer, even in the presence of the non-sensitizing OAc^−^. A split of the very intense ^5^ *D*_0_→^7^ *F*_2_ emission suggests that the Eu^III^ ion is not located at a site with a center of symmetry (Binnemans, 2015 ▸), which is consistent with its distorted tricapped trigonal–prismatic coordination geometry. The split of the ^5^ *D*_0_→^7^ *F*_4_ emission, on the other hand, should be due to the ligand-field effect (Gupta *et al.*, 2015 ▸; Okayasu & Yuasa, 2021 ▸; Puntus *et al.*, 2010 ▸).

## 5. Database survey

A search of the CSD database (CSD version 5.43, update of November 2021; Groom *et al.*, 2016 ▸) using the ConQuest software (version 2021.3.0; Bruno *et al.*, 2002 ▸), found 115 structures of lanthanide compounds including phth^2−^. In six of these structures, phth^2−^ adopts the same μ_3_-η^1^:η^2^:η^2^:η^1^ mode of coordination as in the title compound. This mode of coordination apparently promotes the formation of a one-dimensional coordination framework, as, for example, in [Pr_3_(phen)_2_(phth)_4_(NO_3_)]·H_2_O (phen = 1,10-phenanthroline) (refcode: LAXWOX; Thirumurugan & Natarajan, 2005 ▸), [Nd(Nphgly)(phth)(H_2_O)]·2H_2_O (Nphgly = *N*-phthaloylglycine) (refcode: TOHJEH; Yang *et al.*, 2014 ▸), and [Gd_2_Ni(2,5-pdc)_2_(phth)_2_(H_2_O)_4_]·8H_2_O (2,5-H_2_pdc = 2,5-pyridinedi­carb­oxy­lic acid) (refcode: XOZYER; Mahata *et al.*, 2009 ▸).

Regarding OAc^−^, there are 566 structures containing this deposited in the CSD, none of which also contains phth^2−^. There are, however, structures containing both OAc^−^ and isophthalate (iso-phth^2−^), *e.g*. [Sm_2_(iso-phth)_2_(OAc)_2_(H_2_O)_4_]·H_2_O (refcode: VOJNAK; Jin *et al.*, 2008 ▸), and [Dy_4_(iso-phth)_4_(OAc)_4_(H_2_O)_8_]·2H_2_O (refcode: DIBZEU; Hu *et al.*, 2007 ▸).

## 6. Synthesis and crystallization

All chemicals used in this work were obtained commercially and used without purification: Eu_2_O_3_ (Strategic Elements, 99.99%), phthalic acid (H_2_phth; C_8_H_6_O_4_, BDH laboratory, 99%), NaOH (RCI Labscan, 99.0%), glacial acetic acid (AcOH; CH_3_COOH, QRëC, 99.8%), tetra­hydro­furan (THF; C_4_H_8_O, RCI Labscan, 99.8%), ethanol (EtOH; C_2_H_5_OH, RCI Labscan, 99.7%). Eu(OAc)_3_·4H_2_O, was prepared by dissolving Eu_2_O_3_ (2.5000 g, 7.1038 mmol) in 50.0 mL of deionized water with a few drops of glacial acetic acid (HOAc). After the pH of the suspension was adjusted to 3 using HOAc, the mixture was gently heated and a colorless homogeneous solution was attained. The white powder of Eu(OAc)_3_·4H_2_O was then recovered through slow evaporation of the solvent.

To synthesize the title compound, Eu(OAc)_3_·4H_2_O (0.16 g, 0.40 mmol) was dissolved in 2.0 mL of deionized water to prepare solution **A**. Solution **B** was separately prepared by dissolving Na_2_phth (84 mg, 0.40 mmol) and NaOAc (33 mg, 0.4 mmol) in a mixed solvent prepared from 1.0 mL of deionized water and 5.0 mL of tetra­hydro­furan (THF). Solutions **A** and **B** were then mixed in a 15 mL glass vial. The volume of the reaction was adjusted to 10.0 mL using deionized water and the pH of the solution was adjusted to 4 using HOAc. The reaction was left under stirring at room temperature for 2 h, after which the solvent was slowly evaporated, leading to the crystallization of colorless block-shaped crystals of [Eu(phth)(OAc)(H_2_O)] (78% yield based on Eu^III^). The crystals were characterized using FT–IR spectroscopy (PerkinElmer/Frontier FT–IR instrument; ATR mode; cm^−1^): 3541(*br*), 3419(*br*), 2978(*w*), 1548(*w*), 1402(*m*), 1373(*m*), 804(*s*), 754(*s*), 707(*s*), 650(*s*), 543(*m*), 503(*m*). The room-temperature photoluminescent spectrum was collected using a ASEQ LR-1T broad-range spectrophotometer equipped with an Ultrafire G60 UV LED Flashlight Torch excitation source (5 W, 370 nm)

## 7. Refinement

Crystal data, data collection and structure refinement details are summarized in Table 3 ▸. All H atoms were positioned geometrically and refined isotropically using a riding model. The C—H bond lengths in the aromatic phth^2−^ linker and in OAc^−^ were restrained to 0.93 Å [*U*_iso_(H) = 1.2*U*_eq_(C)] and 0.96 Å [*U*_iso_(H) = 1.5*U*_eq_(C)], respectively. The O—H bond lengths in the coordinated water mol­ecule were restrained to 0.85 Å with *U*_iso_(H) = 1.5*U*_eq_(O).

## Supplementary Material

Crystal structure: contains datablock(s) I. DOI: 10.1107/S2056989022004339/zn2019sup1.cif

Structure factors: contains datablock(s) I. DOI: 10.1107/S2056989022004339/zn2019Isup2.hkl

CCDC reference: 2168116

Additional supporting information:  crystallographic information; 3D view; checkCIF report

## Figures and Tables

**Table 1:** Selected bond lengths (Å)

Eu1—O1^i^	2.570 (2)	Eu1—O4^ii^	2.605 (2)
Eu1—O1	2.397 (2)	Eu1—O5	2.352 (2)
Eu1—O2^i^	2.474 (2)	Eu1—O6^i^	2.434 (3)
Eu1—O3	2.381 (2)	Eu1—O7	2.446 (2)
Eu1—O3^ii^	2.484 (2)		

Symmetry codes: (i) 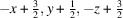 ; (ii) 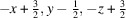 .

**Table 2:** Hydrogen-bond geometry (Å, °)

*D*—H⋯*A*	*D*—H	H⋯*A*	*D*⋯*A*	*D*—H⋯*A*
O7—H7*A*⋯O4^iii^	0.85	2.17	2.9384	149
O7—H7*B*⋯O6^iv^	0.85	2.28	3.0438	150
C3—H3⋯O2	0.93	2.46	2.7741	100

Symmetry codes: (iii) 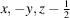 ; (iv) 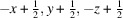 .

**Table 3:** Experimental details

Crystal data	
Chemical formula	[Eu(C_8_H_4_O_4_)(CH_3_O_2_)(H_2_O)]
*M*_r_	393.13
Crystal system, space group	Monoclinic, *C*2/*c*
Temperature (K)	293
*a*, *b*, *c* (Å)	26.5184 (15), 7.2632 (2), 15.3622 (8)
β (°)	130.906 (9)
*V* (Å^3^)	2236.3 (3)
*Z*	8
Radiation type	Mo *K*α
μ (mm^−1^)	5.63
Crystal size (mm)	0.2 × 0.1 × 0.1

Data collection	
Diffractometer	Rigaku SuperNova, single source at offset/far, HyPix3000
Absorption correction	Multi-scan (*CrysAlis PRO*; Rigaku OD, 2019 ▸)
*T*_min_, *T*_max_	0.218, 1.000
No. of measured, independent and observed [*I* > 2σ(*I*)] reflections	9923, 2393, 2138
*R*_int_	0.032
(sin θ/λ)_max_ (Å^−1^)	0.648

Refinement	
*R*[*F* ^2^ > 2σ(*F* ^2^)], *wR*(*F* ^2^), *S*	0.024, 0.052, 1.05
No. of reflections	2393
No. of parameters	167
No. of restraints	1
H-atom treatment	H-atom parameters constrained
Δρ_max_, Δρ_min_ (e Å^−3^)	0.64, −0.79

Computer programs: *CrysAlis PRO* (Rigaku OD, 2019 ▸), *SHELXT2018/2* (Sheldrick, 2015*a* ▸), *SHELXL2018/3* (Sheldrick, 2015*b* ▸) and *OLEX2* (Dolomanov *et al.*, 2009 ▸).

## supplementary crystallographic information

### Crystal data

**Table:**

[Eu(C_8_H_4_O_4_)(CH_3_O_2_)(H_2_O)]	*F*(000) = 1504
*M**_r_* = 393.13	*D*_x_ = 2.335 Mg m^−^^3^
Monoclinic, *C*2/*c*	Mo *K*α radiation, λ = 0.71073 Å
*a* = 26.5184 (15) Å	Cell parameters from 7078 reflections
*b* = 7.2632 (2) Å	θ = 2.0–27.4°
*c* = 15.3622 (8) Å	µ = 5.63 mm^−^^1^
β = 130.906 (9)°	*T* = 293 K
*V* = 2236.3 (3) Å^3^	Block, clear light colourless
*Z* = 8	0.2 × 0.1 × 0.1 mm

### Data collection

**Table:**

Rigaku SuperNova, Single source at offset/far, HyPix3000 diffractometer	2393 independent reflections
Radiation source: micro-focus sealed X-ray tube, SuperNova (Mo) X-ray Source	2138 reflections with *I* > 2σ(*I*)
Mirror monochromator	*R*_int_ = 0.032
Detector resolution: 10.0000 pixels mm^-1^	θ_max_ = 27.4°, θ_min_ = 2.0°
ω scans	*h* = −33→33
Absorption correction: multi-scan (CrysAlisPro; Rigaku OD, 2019)	*k* = −9→9
*T*_min_ = 0.218, *T*_max_ = 1.000	*l* = −19→18
9923 measured reflections	

### Refinement

**Table:**

Refinement on *F*^2^	Primary atom site location: dual
Least-squares matrix: full	Hydrogen site location: mixed
*R*[*F*^2^ > 2σ(*F*^2^)] = 0.024	H-atom parameters constrained
*wR*(*F*^2^) = 0.052	*w* = 1/[σ^2^(*F*_o_^2^) + (0.0235*P*)^2^ + 1.2859*P*] where *P* = (*F*_o_^2^ + 2*F*_c_^2^)/3
*S* = 1.05	(Δ/σ)_max_ = 0.001
2393 reflections	Δρ_max_ = 0.64 e Å^−^^3^
167 parameters	Δρ_min_ = −0.79 e Å^−^^3^
1 restraint	

### Special details

**Table:**

Geometry. All esds (except the esd in the dihedral angle between two l.s. planes) are estimated using the full covariance matrix. The cell esds are taken into account individually in the estimation of esds in distances, angles and torsion angles; correlations between esds in cell parameters are only used when they are defined by crystal symmetry. An approximate (isotropic) treatment of cell esds is used for estimating esds involving l.s. planes.

### Fractional atomic coordinates and isotropic or equivalent isotropic displacement parameters (Å^2^)

**Table:**

	*x*	*y*	*z*	*U*_iso_*/*U*_eq_	
Eu1	0.75770 (2)	0.36927 (2)	0.71208 (2)	0.01709 (7)	
O1	0.69745 (10)	0.1993 (3)	0.7522 (2)	0.0219 (5)	
O4	0.68510 (12)	0.6542 (3)	0.8366 (2)	0.0264 (6)	
O3	0.68174 (10)	0.5756 (3)	0.69468 (19)	0.0185 (5)	
O6	0.69030 (12)	−0.0954 (3)	0.5869 (2)	0.0290 (6)	
O2	0.62648 (11)	−0.0281 (3)	0.6844 (2)	0.0273 (6)	
O7	0.72235 (14)	0.5336 (4)	0.5410 (2)	0.0422 (7)	
H7A	0.697069	0.483288	0.474914	0.063*	
H7B	0.708422	0.643920	0.528604	0.063*	
O5	0.67155 (11)	0.2044 (3)	0.5460 (2)	0.0308 (6)	
C7	0.59568 (15)	0.4602 (4)	0.6922 (3)	0.0185 (7)	
C2	0.58415 (15)	0.2718 (4)	0.6633 (3)	0.0185 (7)	
C8	0.65937 (15)	0.5624 (4)	0.7479 (3)	0.0181 (7)	
C9	0.66224 (16)	0.0380 (5)	0.5165 (3)	0.0257 (8)	
C1	0.63844 (17)	0.1403 (4)	0.7002 (3)	0.0196 (8)	
C10	0.61510 (19)	−0.0007 (5)	0.3902 (3)	0.0412 (10)	
H10A	0.616586	−0.129448	0.377789	0.062*	
H10B	0.627783	0.070141	0.354452	0.062*	
H10C	0.570626	0.032152	0.357152	0.062*	
C6	0.54332 (17)	0.5698 (5)	0.6621 (3)	0.0282 (8)	
H6	0.551117	0.692945	0.683999	0.034*	
C3	0.52024 (16)	0.2027 (5)	0.6028 (3)	0.0257 (8)	
H3	0.512351	0.077936	0.585067	0.031*	
C4	0.46810 (17)	0.3156 (5)	0.5685 (3)	0.0301 (9)	
H4	0.425093	0.268326	0.524046	0.036*	
C5	0.47994 (17)	0.4983 (5)	0.6002 (3)	0.0318 (9)	
H5	0.445276	0.573408	0.579933	0.038*	

### Atomic displacement parameters (Å^2^)

**Table:**

	*U*^11^	*U*^22^	*U*^33^	*U*^12^	*U*^13^	*U*^23^
Eu1	0.01881 (11)	0.01068 (11)	0.02213 (12)	0.00003 (6)	0.01356 (9)	−0.00015 (6)
O1	0.0190 (12)	0.0157 (11)	0.0322 (14)	0.0006 (10)	0.0173 (11)	0.0032 (11)
O4	0.0294 (14)	0.0245 (14)	0.0270 (14)	−0.0045 (10)	0.0191 (12)	−0.0049 (11)
O3	0.0205 (12)	0.0112 (11)	0.0261 (13)	−0.0006 (9)	0.0162 (11)	0.0012 (10)
O6	0.0311 (14)	0.0253 (14)	0.0243 (14)	0.0014 (11)	0.0154 (12)	0.0038 (11)
O2	0.0255 (13)	0.0127 (12)	0.0439 (15)	−0.0017 (10)	0.0228 (12)	−0.0027 (11)
O7	0.0635 (19)	0.0265 (15)	0.0297 (15)	−0.0021 (14)	0.0275 (15)	0.0026 (12)
O5	0.0299 (14)	0.0218 (13)	0.0312 (14)	−0.0048 (11)	0.0158 (12)	−0.0053 (11)
C7	0.0202 (17)	0.0164 (17)	0.0204 (17)	0.0005 (13)	0.0139 (15)	0.0027 (14)
C2	0.0191 (17)	0.0157 (17)	0.0214 (17)	0.0006 (13)	0.0136 (15)	0.0012 (14)
C8	0.0174 (16)	0.0097 (16)	0.0256 (18)	0.0063 (13)	0.0135 (15)	0.0054 (14)
C9	0.0231 (18)	0.028 (2)	0.0270 (19)	−0.0079 (16)	0.0168 (17)	−0.0048 (17)
C1	0.0243 (19)	0.0166 (18)	0.0210 (19)	0.0015 (13)	0.0161 (16)	0.0041 (13)
C10	0.049 (3)	0.037 (2)	0.023 (2)	−0.0107 (19)	0.017 (2)	−0.0014 (18)
C6	0.029 (2)	0.0200 (18)	0.041 (2)	0.0016 (16)	0.0257 (18)	0.0003 (17)
C3	0.0239 (18)	0.0208 (18)	0.0279 (19)	−0.0018 (15)	0.0149 (16)	0.0013 (16)
C4	0.0168 (18)	0.033 (2)	0.035 (2)	−0.0030 (16)	0.0143 (17)	0.0025 (18)
C5	0.0228 (19)	0.027 (2)	0.044 (2)	0.0075 (15)	0.0213 (18)	0.0055 (18)

### Geometric parameters (Å, º)

**Table:**

Eu1—O1^i^	2.570 (2)	Eu1—O4^ii^	2.605 (2)
Eu1—O1	2.397 (2)	Eu1—O5	2.352 (2)
Eu1—O2^i^	2.474 (2)	Eu1—O6^i^	2.434 (3)
Eu1—O3	2.381 (2)	Eu1—O7	2.446 (2)
Eu1—O3^ii^	2.484 (2)		
			
O1—Eu1—Eu1^i^	100.62 (6)	Eu1—O3—Eu1^i^	107.20 (8)
O1^i^—Eu1—Eu1^i^	36.44 (5)	C8—O3—Eu1^i^	95.16 (19)
O1—Eu1—O1^i^	135.94 (6)	C8—O3—Eu1	126.05 (19)
O1—Eu1—O4^ii^	112.11 (8)	C9—O6—Eu1^ii^	133.7 (2)
O1^i^—Eu1—O4^ii^	110.21 (7)	C1—O2—Eu1^ii^	96.9 (2)
O1—Eu1—O3^ii^	72.40 (7)	Eu1—O7—H7A	121.4
O1—Eu1—O6^i^	69.36 (8)	Eu1—O7—H7B	120.3
O1—Eu1—O2^i^	138.23 (8)	H7A—O7—H7B	104.5
O1—Eu1—O7	132.57 (9)	C9—O5—Eu1	134.6 (2)
O4^ii^—Eu1—Eu1^i^	146.65 (5)	C2—C7—C8	126.3 (3)
O3—Eu1—Eu1^i^	37.29 (5)	C6—C7—C2	119.2 (3)
O3^ii^—Eu1—Eu1^i^	141.16 (5)	C6—C7—C8	114.4 (3)
O3—Eu1—O1^i^	71.11 (7)	C7—C2—C1	123.0 (3)
O3—Eu1—O1	72.28 (8)	C3—C2—C7	118.7 (3)
O3^ii^—Eu1—O1^i^	130.07 (7)	C3—C2—C1	118.3 (3)
O3^ii^—Eu1—O4^ii^	51.12 (7)	O4—C8—Eu1^i^	63.93 (18)
O3—Eu1—O4^ii^	162.55 (8)	O4—C8—O3	120.3 (3)
O3—Eu1—O3^ii^	141.75 (4)	O4—C8—C7	119.7 (3)
O3—Eu1—O6^i^	79.11 (8)	O3—C8—Eu1^i^	58.54 (16)
O3—Eu1—O2^i^	119.05 (7)	O3—C8—C7	119.3 (3)
O3—Eu1—O7	82.61 (9)	C7—C8—Eu1^i^	156.5 (2)
O6^i^—Eu1—Eu1^i^	66.91 (5)	O6—C9—C10	119.2 (3)
O6^i^—Eu1—O1^i^	80.35 (8)	O5—C9—O6	124.1 (3)
O6^i^—Eu1—O4^ii^	118.34 (8)	O5—C9—C10	116.7 (3)
O6^i^—Eu1—O3^ii^	75.15 (8)	O1—C1—Eu1^ii^	62.34 (16)
O6^i^—Eu1—O2^i^	73.70 (8)	O1—C1—C2	120.2 (3)
O6^i^—Eu1—O7	144.26 (8)	O2—C1—Eu1^ii^	57.86 (16)
O2^i^—Eu1—Eu1^i^	81.77 (5)	O2—C1—O1	120.1 (3)
O2^i^—Eu1—O1^i^	51.37 (7)	O2—C1—C2	119.6 (3)
O2^i^—Eu1—O4^ii^	69.76 (7)	C2—C1—Eu1^ii^	174.1 (2)
O2^i^—Eu1—O3^ii^	79.99 (7)	C9—C10—H10A	109.5
O7—Eu1—Eu1^i^	79.97 (7)	C9—C10—H10B	109.5
O7—Eu1—O1^i^	64.69 (8)	C9—C10—H10C	109.5
O7—Eu1—O4^ii^	82.51 (9)	H10A—C10—H10B	109.5
O7—Eu1—O3^ii^	133.38 (9)	H10A—C10—H10C	109.5
O7—Eu1—O2^i^	89.12 (9)	H10B—C10—H10C	109.5
O5—Eu1—Eu1^i^	125.35 (6)	C7—C6—H6	119.5
O5—Eu1—O1	71.29 (8)	C5—C6—C7	120.9 (3)
O5—Eu1—O1^i^	133.75 (8)	C5—C6—H6	119.5
O5—Eu1—O4^ii^	73.60 (8)	C2—C3—H3	119.3
O5—Eu1—O3	92.82 (8)	C4—C3—C2	121.3 (3)
O5—Eu1—O3^ii^	89.44 (7)	C4—C3—H3	119.3
O5—Eu1—O6^i^	140.44 (9)	C3—C4—H4	120.1
O5—Eu1—O2^i^	139.98 (8)	C5—C4—C3	119.8 (3)
O5—Eu1—O7	70.49 (9)	C5—C4—H4	120.1
Eu1—O1—Eu1^ii^	104.00 (8)	C6—C5—H5	120.0
C1—O1—Eu1^ii^	91.53 (18)	C4—C5—C6	119.9 (3)
C1—O1—Eu1	139.6 (2)	C4—C5—H5	120.0
C8—O4—Eu1^i^	90.7 (2)		
			
Eu1—O1—C1—Eu1^ii^	113.9 (3)	C7—C2—C3—C4	1.7 (5)
Eu1^ii^—O1—C1—O2	−3.0 (3)	C7—C6—C5—C4	0.7 (6)
Eu1—O1—C1—O2	110.9 (3)	C2—C7—C8—Eu1^i^	140.5 (5)
Eu1^ii^—O1—C1—C2	174.0 (3)	C2—C7—C8—O4	−127.1 (3)
Eu1—O1—C1—C2	−72.1 (4)	C2—C7—C8—O3	62.7 (4)
Eu1^i^—O4—C8—O3	16.5 (3)	C2—C7—C6—C5	−2.9 (5)
Eu1^i^—O4—C8—C7	−153.6 (2)	C2—C3—C4—C5	−3.9 (5)
Eu1—O3—C8—Eu1^i^	115.7 (2)	C8—C7—C2—C1	8.9 (5)
Eu1—O3—C8—O4	98.2 (3)	C8—C7—C2—C3	−174.4 (3)
Eu1^i^—O3—C8—O4	−17.4 (3)	C8—C7—C6—C5	173.7 (3)
Eu1—O3—C8—C7	−91.6 (3)	C1—C2—C3—C4	178.4 (3)
Eu1^i^—O3—C8—C7	152.8 (2)	C6—C7—C2—C1	−174.9 (3)
Eu1^ii^—O6—C9—O5	21.5 (5)	C6—C7—C2—C3	1.7 (5)
Eu1^ii^—O6—C9—C10	−158.7 (3)	C6—C7—C8—Eu1^i^	−35.8 (7)
Eu1^ii^—O2—C1—O1	3.1 (3)	C6—C7—C8—O4	56.7 (4)
Eu1^ii^—O2—C1—C2	−173.9 (3)	C6—C7—C8—O3	−113.6 (3)
Eu1—O5—C9—O6	23.5 (5)	C3—C2—C1—O1	177.3 (3)
Eu1—O5—C9—C10	−156.3 (3)	C3—C2—C1—O2	−5.7 (5)
C7—C2—C1—O1	−6.1 (5)	C3—C4—C5—C6	2.7 (5)
C7—C2—C1—O2	170.9 (3)		

Symmetry codes: (i) −*x*+3/2, *y*+1/2, −*z*+3/2; (ii) −*x*+3/2, *y*−1/2, −*z*+3/2.

### Hydrogen-bond geometry (Å, º)

**Table:**

*D*—H···*A*	*D*—H	H···*A*	*D*···*A*	*D*—H···*A*
O7—H7*A*···O4^iii^	0.85	2.17	2.9384	149
O7—H7*B*···O6^iv^	0.85	2.28	3.0438	150
C3—H3···O2	0.93	2.46	2.7741	100

Symmetry codes: (iii) *x*, −*y*, *z*−1/2; (iv) −*x*+1/2, *y*+1/2, −*z*+1/2.
